# Carrageenan maintains the contractile phenotype of vascular smooth muscle cells by increasing macromolecular crowding in vitro

**DOI:** 10.1186/s40001-024-01843-2

**Published:** 2024-04-22

**Authors:** Qing Liu, Hong-Jing Jiang, Yin-Di Wu, Jian-Dong Li, Xu-Heng Sun, Cong Xiao, Jian-Yi Xu, Zhan-Yi Lin

**Affiliations:** 1 Ji Hua Institute of Biomedical Engineering Technology, Ji Hua Laboratory, Foshan, Guangdong China; 2 School of Medicine, South China University of Technology, Guangzhou, Guangdong China; 3 Department of Cardiology, Guangdong Cardiovascular Institute, Guangdong Provincial People’s Hospital (Guangdong Academy of Medical Sciences), Southern Medical University, Guangzhou, Guangdong China

**Keywords:** Carrageenan, Macromolecular crowding, Vascular smooth muscle cells, Contractile phenotype, Transcriptomics

## Abstract

**Background:**

The contractile phenotype of vascular smooth muscle cells (VSMCs) results in good diastolic and contractile capacities, and its altered function is the main pathophysiological basis for diseases such as hypertension. VSMCs exist as a synthetic phenotype in vitro, making it challenging to maintain a contractile phenotype for research. It is widely recognized that the common medium in vitro is significantly less crowded than in the in vivo environment. Additionally, VSMCs have a heightened sense for detecting changes in medium crowding. However, it is unclear whether macromolecular crowding (MMC) helps maintain the VSMCs contractile phenotype.

**Purpose:**

This study aimed to explore the phenotypic, behavioral and gene expression changes of VSMCs after increasing the crowding degree by adding carrageenan (CR).

**Methods:**

The degree of medium crowding was examined by a dynamic light scattering assay; VSMCs survival and activity were examined by calcein/PI cell activity and toxicity and CCK-8 assays; VSMCs phenotypes and migration were examined by WB and wound healing assays; and gene expression was examined by transcriptomic analysis and RT-qPCR.

**Results:**

Notably, 225 μg/mL CR significantly increased the crowding degree of the medium and did not affect cell survival. Simultaneously, CR significantly promoted the contraction phenotypic marker expression in VSMCs, shortened cell length, decreased cell proliferation, and inhibited cell migration. CR significantly altered gene expression in VSMCs. Specifically, 856 genes were upregulated and 1207 genes were downregulated. These alterations primarily affect the cellular ion channel transport, microtubule movement, respiratory metabolism, amino acid transport, and extracellular matrix synthesis. The upregulated genes were primarily involved in the cytoskeleton and contraction processes of VSMCs, whereas the downregulated genes were mainly involved in extracellular matrix synthesis.

**Conclusions:**

The in vitro study showed that VSMCs can maintain the contractile phenotype by sensing changes in the crowding of the culture environment, which can be maintained by adding CR.

**Graphical Abstract:**

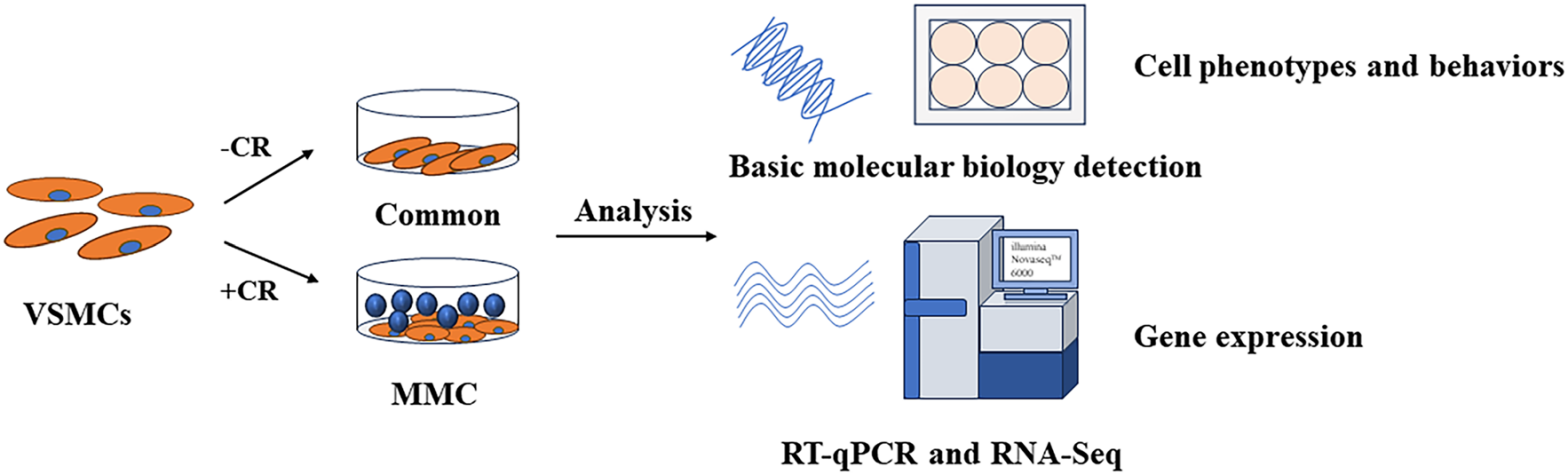

## Background

Vascular smooth muscle cells (VSMCs) are specialized cells in the middle layer of blood vessels that express various proteins associated with contractile function. They are phenotypically plastic and play a critical role in vascular structural integrity, regulation of blood pressure, and vascular remodeling [1]. Phenotypic transition of VSMCs is a crucial factor in the early stages of various cardiovascular diseases, such as hypertension and atherosclerosis [2]. VSMCs constantly differentiate from the contractile state to other SMC-like phenotypes. This process involves a decrease or loss of contractility-associated protein expression and an increase in extracellular matrix protein production [1]. The phenotypic transition of VSMCs affects their proliferation, migration, and contractile functions [3]. Therefore, it is essential to precisely control these phenotypes [4]. The use of carrageenan (CR) as a macromolecular crowding (MMC) agent to maintain VSMC contraction phenotype has not been extensively investigated. VSMCs are typically cultured in vitro in two-dimensional (2D) cell culture plates. However, the traditional 2D cell cultures only partially recapitulate the cellular environment, which differs significantly from the in vivo environment. The absence of a complex and abundant tissue-specific extracellular matrix can cause cellular phenotype and function loss, as well as epigenetic drift [5]. To address these deficiencies, numerous studies have been conducted to create bionic microenvironments that regulate cellular phenotypic behavior and function. These studies have explored various aspects, including three-dimensional culture [6], mechanical cues [7], oxygen concentration [8], growth factors [9], and surface morphology [10]. However, the regulation of the VSMCs contractile phenotype, behavior, and function by MMC remains unexplored.

In eukaryotic cell cultures, cells grow in a crowded environment in vivo. However, the common in vitro medium contains only 4–16 g/L macromolecules supplemented with 5–20% fetal bovine serum (FBS). This is much lower than that in vivo and can cause cells to undergo epigenetic changes, leading to altered protein and gene profiles, phenotypic drift, loss of function, and senescence. Consequently, it does not provide an ideal cellular model for vascular disease research, such as hypertension [5, 11]. The addition of inert polydisperse macromolecules to the culture medium can simulate an in vivo crowding environment [12]. Several studies have used MMC reagents such as Ficoll [13], dextran sulfate (D × S) [14], and CR [15] to increase medium crowding and modulate the biological behaviors and functions of cells, such as myofibroblast phenotype, MSCs behavior, and gene and protein expression [16–18]. Different microenvironments may cause adaptive changes in cells, and different MMC, cell types, and culture conditions may have varying effects. Therefore, it is unclear whether medium crowding helps in maintaining the contractile phenotype of VSMCs.

Most in vitro studies on VSMCs phenotypic shifts have focused on gene expression, migration, and cell proliferation [19]. Transcriptomic sequencing technology offers a solution for whole-gene testing to explore changes in gene expression [20]. Ramalingam et al. detected changes in gene expression in human gingival fibroblasts by adding Ficoll70/400 using RT-qPCR [18]. Similarly, Tsiapalis et al. detected changes in gene expression in equine tendon cells under hypoxia and CR using RT-qPCR [21]. Previous studies have identified only a limited number of target gene changes and failed to provide a comprehensive characterization of genomic changes. However, researchers can comprehensively assess all the molecules and signaling pathways associated with cellular phenotypes and behaviors by detecting the expression of all genes. This provides molecular information about MMC in VSMCs phenotypes and behaviors, which is beneficial for providing an ideal cellular model for hypertensive disease studies [22]. CR is a natural polysaccharide with a structure similar to that of the glycosaminoglycans (GAGs) found in natural tissues. It is highly negatively charged, polydispersible, and can be effectively dispersed in the culture medium. This is one of the most effective MMC available [23]. Therefore, in this study, we combined a common medium with CR to comprehensively assess the altered VSMCs phenotypes, behaviors, and gene expression after the addition of CR using protein analysis and transcriptome sequencing. We aimed to gain a comprehensive understanding of the alterations in VSMCs phenotypes and behaviors by adding MMC, which can provide a cellular model for the pathogenesis of vascular diseases such as hypertension.

## Materials and methods

### Isolation and culture of VSMCs

Cells were extracted from healthy bovine aorta mesentery after obtaining appropriate animal ethical approvals. The bovine aorta mesentery is a part of the bovine aorta that is left behind after the removal of the inner and outer membranes and is mainly composed of VSMCs and an elastin layer. The connective tissue around the aorta was removed and the inner and outer membranes were peeled off. The aortic mesentery was cut into small pieces. Subsequently, the mid-aortic membrane was affixed to T75 plastic culture flasks and incubated at 37 °C with 5% CO_2_. The T75 plastic culture flasks were inverted so that the side with the mesentery tissue block faced upwards and remained attached for 4 h. Then, the flasks were turned over and 15 mL of culture medium containing DMEM F-12 (Gibco, USA), 10% FBS (Corning, USA), and 1% penicillin–streptomycin (Gibco, USA) was added. After 7 days, the first VSMCs grew around the aortic mesentery. Once the cells reached 80–90%, they were treated with 0.25% trypsin (Gibco, USA) and passaged in cell culture plates. The experiment was approved by the Medical Ethics Committee of Guangdong Provincial People's Hospital (Guangdong Provincial Academy of Medical Sciences; reference number: KY2023-192-01).

### MMC construction

VSMCs were inoculated into cell culture plates at 2.0 × 10^4^ cells/cm^2^. After 24 h, the original medium was replaced with MMC agent. The MMC agent was 225 μg/mL CR (Sigma Aldrich, USA). The medium was changed every 2–3 d. All cells were used at passages 3–5, and samples were analyzed on day 5.

### Immunocytochemistry

The cell layer was washed with phosphate buffer solution (PBS, Sigma-Aldrich, USA) and fixed with 4% paraformaldehyde (Sigma-Aldrich, USA) for 30 min at room temperature. After three washes with PBS, the cells were permeabilized with 0.25% Triton X-100 for 20 min. Non-specific sites were blocked with 3% bovine serum albumin (Sigma Aldrich, USA) for 30 min. The cells were incubated with a primary antibody overnight at 4 °C. Antibodies used were α-SMA (1:200, NBP1-30,894, Novus Biologicals, USA), calponin (1:200, 13,938–1-AP, Proteintech, USA), and smoothelin (1:200, orb158429, Biorbyt, UK). After three washes in PBS, α-SMA, calponin, and smoothelin were incubated with goat anti-rabbit antibodies; Dylight488 and Dylight596, respectively. The nuclei were stained with DAPI. Images were captured using an Olympus IX-81 inverted fluorescence microscope (Olympus IX-81, Olympus Corporation, Japan).

### Dynamic light scattering (DLS) measurements

According to previous studies [23, 24], DLS characterizes the particle size in solution and can be used to detect the charge, particle size, and polydispersity coefficients of MMC reagents. The study examined the hydrodynamic radius (R_H_) and volume occupancy fraction (FVO) of 25 and 225 μg/mL CR using Malvern particle sizer (Zetasizer Ultra, Malvern Instruments, UK). Crowding solutions were prepared in PBS to simulate physiological conditions. FVO was calculated based on the R_H_ values as described in the literature [25].

Calculations for estimating the fractional volume occupancy are as follows:

1 $$\bullet \qquad {\text{The volume of a sphere }} = { 4}/{3 }\pi {\text{r}}^{{3}} = { 4}/{3 }\pi \, \left( {{\text{R}}_{{\text{H}}} } \right)^{{3}}$$

- The MW of carrageenan is ~ 550,000 Da.
  The number of carrageenan molecules present in X mg was calculated as follows:
  2 $$\left( {{\text{X }} \times { 1}0^{{ - {3}}} } \right) \, \times \, \left( {{6}.0{23 } \times { 1}0^{{{23}}} } \right) \, \div { 55}0,000$$
- The volume occupied by these number of CR molecules in 1 mL was calculated by multiplying 1 and 2 and then expressed in percentage to estimate the fraction volume occupancy:
  $${\text{FVO }}\% \, =\,{ 4}/{3 }\pi \, \left( {{\text{R}}_{{\text{H}}} } \right)^{{3}} \times \, \left( {{\text{X }} \times { 1}0^{{ - {3}}} } \right) \, \times \, \left( {{6}.0{23 } \times { 1}0^{{{23}}} } \right) \, \div { 55}0,000.$$

### Calcein/PI cell activity and cytotoxicity assay

Cell survival was assessed on days three and five using a calcein/PI cell activity and cytotoxicity assay kit (Beyotime Biotechnology). After removing the medium from the 96-well plates, cells were washed with PBS. Subsequently, they were stained with 100 μL of calcein AM/PI assay workup and incubated at 37 ℃ away from light for 30 min. The staining was observed under an inverted fluorescence microscope (Olympus IX-81, Olympus Corporation, Japan), with green fluorescence indicating calcein AM and red fluorescence indicating PI.

### Western blotting

After 5 days of culture, the cells were collected and washed three times with pre-cooled PBS. The cells were lysed using RIPA lysis buffer (Beyotime Biotechnology, China) and protease inhibitors (Thermo Fisher, USA) on ice for 30 min. The lysate was centrifuged at 12,000 rpm and 4 ℃ for 15 min, and the supernatant was collected. The protein concentration in each sample was determined using a BCA assay. Protein samples were separated using 10% SDS-PAGE gels with 15 μg per well, electro-transferred to nitrocellulose membranes, and blocked with milk. The membranes were then incubated with α-SMA (1:2000, Rabbit polyclonal, NBP1-30,894, Novusbio, USA), CNN 1 (1:2000, Rabbit polyclonal, 13,938–1-AP, Proteintech, USA), and anti-histone 3 (1:2000, Rabbit polyclonal, 4499 s, Cell signaling, USA). The protein samples were incubated with goat anti-rabbit conjugated horseradish peroxidase (1:20,000; Abbkine, China). Blots were developed using an enhanced chemiluminescence method according to the manufacturer’s protocol (Biosharp, China).

### VSMCs contraction assay

VSMCs in the logarithmic growth stage were inoculated in 12-well plates at a density of 10,000 cells per well. Next, 1 mL of the medium was added to each well. After 24 h, the medium was replaced with CR or no-CR medium. After 5 days, the medium was discarded, and 4% PFA was added for termination. The cells were then subjected to staining with CoraLite^®^594-Phalloidin (PF00003, Proteintech, USA) and imaged using an inverted fluorescence microscope (Olympus IX-81 Olympus Corporation, Japan). The length of 80 cells in each group was measured using ImageJ software.

### Cell proliferation assessment

VSMCs proliferation was assessed using Cell Counting Kit-8 (CCK-8) (Dojindo, Japan). VSMCs were seeded in 96-well plates, and the cells were divided into blank, control, and experimental groups. The CCK-8 assay was performed on days 0, 1, 3, and 5. The medium and CCK8 solution were mixed in a 10:1 ratio. Next, 100 μL of the mixture was added to each well and incubated for 4 h at 37 ℃ with 5% CO_2_. Finally, the absorbance was measured at 450 nm using a full-wavelength enzyme labeling instrument.

### Cell migration assessment

VSMCs migration was assessed using the wound healing assay [26]. In a 12-well plate, VMSCs were inoculated and scratches were made with a 200-μL plastic pipette gun tip once they reached 90% confluence. After washing with PBS, the cells were cultured in serum-free medium containing CR and a common serum-free medium. The wound area was imaged using a microscope at 0, 12, 30, and 48 h.

### Transcript sequencing and data analysis

After 5 days of culture, VSMCs were analyzed using reference transcriptome sequencing. RNA was isolated and purified from total samples using TRIzol (Thermo Fisher, USA). Total RNA amount and purity were assessed using NanoDrop ND-1000 (NanoDrop, Wilmington, DE, USA). The RNA integrity was evaluated using Bioanalyzer 2100 (Agilent, CA, USA). The concentration was > 50 ng/μL, and the RIN value was > 7.0. A total RNA amount of > 1 μg was deemed sufficient for downstream experiments. The library was fragmented with divalent cations at high temperatures, followed by polymerase chain reaction to construct a library with a fragment size of 300–50 bP. Bipartite sequencing was performed using an Illumina Novaseq™ PE6000 (LC-Bio Technology CO., Ltd., Hangzhou, China) with PE150 as the sequencing mode. After transcriptome sequencing using the Illumina paired-end RNA-seq method, raw data were subjected to quality control using FastQC (0.10.1, https://github.com/OpenGene/fastp) software to remove junctions, repetitive sequences, and low-quality sequences. Default parameters were used to compare the sequencing data to the genome using HISAT2 (2.2.1, https://ccb.jhu.edu/software/hisat2) and the species was *Bos taurus*. Genes were then assembled and quantified using FPKM (FPKM = [total_exon_/mapped_reads (million) × exon_length (kB)]. The differentially expressed mRNAs were selected with fold change > 2 or fold change < 0.5 and with a parametric F-test comparing nested linear models (*p* value < 0.05) using the R package edge (https://bioconductor.org/packages/release/bioc/html/edgeR.html). Finally, interacting genes clusters and GO and KEGG enrichment analyses were performed using STRING and DAVID software.

### RT-qPCR

RNA was extracted from the VSMCs after 5 days of culture using an RNA extraction kit (Beyotime Biotechnology, China). The concentration and purity of the RNA were then measured. Next, 400 ng of RNA was taken from each sample and used to generate 20 μL of cDNA through reverse transcription using the Reverse Transcription Kit (Yeason, China). Genes were amplified separately using cDNA as a template, with GAPDH serving as an internal reference gene. The two-step PCR amplification standard procedure involved pre-denaturation at 95 ℃ for 5 min in one cycle, followed by PCR translation at 95 ℃ for 10 s and 60 ℃ for 30 s in 40 cycles. The solubilization curve was achieved by heating at 95 ℃ for 15 s, cooling at 60 ℃ for 30 s, and then heating again at 95 ℃ for 15 s. Please refer to Table 1 for the primer sequences.

**Table 1:** Primer sequences for RT-qPCR

Gene	Primer sequences
ACTA2	F: 5′-AAGTACTCTGTCTGGATTGGTG-3′ R: 5′-GTGTCTTAGAAACATTTGCGGT-3′
CNN1	F: 5′-CAAGGCCATCACCAAGTACG-3′ R: 5′-TGGCTCAAATCTCCGTTCCT-3′
TAGLN	F: 5′-ATGTTCCAGACCGTTGACCT-3′ R: 5′-CCTCTTATGCTCCTGGGCTT-3′
GAPDH	F: 5′-GGCGTGAACCACGAGAAGTATAA-3′ R: 5′-CCCTCCACGATGCCAAAGT-3′
TGFB2	F: 5′-AAGCCAGAGTGCC TGAACAAC-3′ R: 5′-TTTCACGACTTTGC TGTCAATGT-3′
TIMP3	F: 5′-CCTTTGGCACGATGGTCTACA-3′ R: 5′-TTAAGGCCACAGAGACTTTCAGAAG-3′
LTBP1	F: 5′-GATTTGGGCCAGATCCTACCT-3′ R: 5′-CGGTAACACGGCCCTTTCT-3′
COL1A1	F: 5′-GCTACTACCGGGCTGATGAT-3′ R: 5′-TTCTCCGCTCTTCCAGTCAG-3′
COL3A1	F: 5′-CTATGGGCATCAAAGGACATCG-3′ R: 5′-CAGATCCTCTTTCACCTCTGTT-3′
FN1	F: 5′-GCGATTGTCTGATTCTGGCTT-3′ R: 5′-TCTCCCTGACGATCCCACTT-3′
MMP1	F: 5′-TGTGGAGACGGTGAAGAAATACCT-3′ R: 5′-AGCTTTTCAGTTATGAGGCCACC-3′
MMP2	F: 5′-CATACAACTTTGACAAGGACGG-3′ R: 5′-TACTTCTTGTCGCGGTCGTAG-3′
MMP3	F: 5′-GGCTGCAAGGGACAAGGA A-3′ R: 5′-CAAACTGTTTCGTATCCTTTGCAA-3′
DCN	F: 5′-ACTTGGCACCAACCCGCTGA-3′ R: 5′-GGGAAGGAGGAAGACCTTGAGGGA-3′

### Statistical analyses

Statistical analysis data are presented as mean ± standard deviation. GraphPad Prism 9.5 software was used for the statistical analysis. All experiments were conducted at least thrice. A non-matching ratio t-test was used for the two groups that followed a normal distribution, and a Chi-square test was used for non-parametric data. A two-way ANOVA test was used to compare multiple groups of data, and a *P*-value of less than 0.05 was considered statistically significant.

## Results

### VSMCs phenotypic identification

**Fig. 1:**
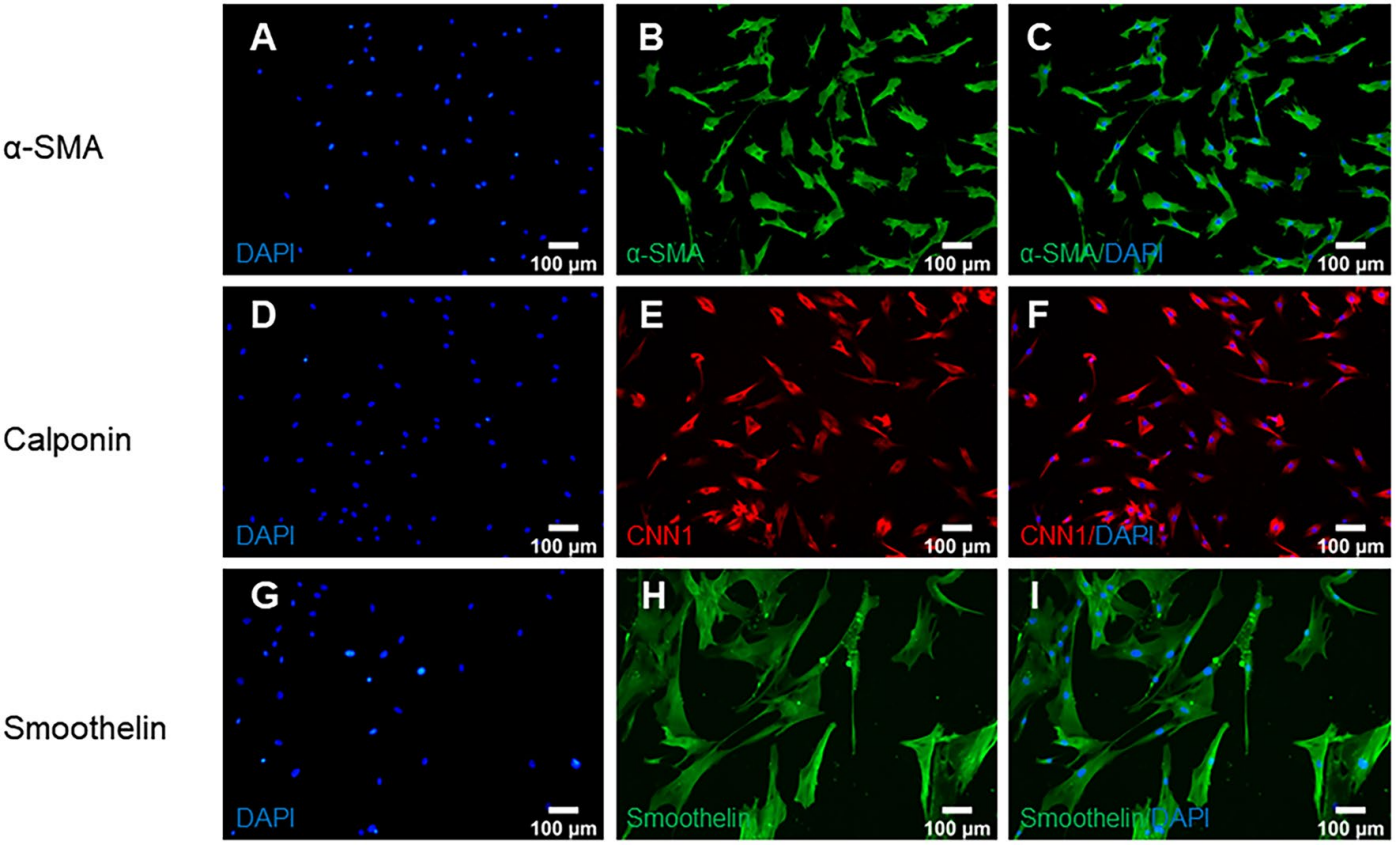
Phenotypic identification of BVSMCs (**A**–**I**). A-C α-SMA fluorescence staining of BVSMCs, α-SMA: green; nucleus: blue; **C** is the merged figure of **A** and **B**; **D**–**F** calponin fluorescence staining, calponin: red; nucleus: blue; F is the merged figure of **D** and **E**; **G**–**I** Smoothelin fluorescence staining, smoothelin: green; nucleus: blue; **I** is the merged figure of G and H; scale bar: 100 μm

First, the VSMCs were characterized to ensure their quality. Immunocytochemistry (ICC) detected the specific markers α-SMA, calponin, and smoothelin of VSMCs (Fig. 1). Green fluorescence was used to stain α-SMA and smoothelin, red fluorescence was used to stain calponin, and blue fluorescence was used to stain the nuclei of the cells. The results indicated that the VSMCs were of high purity, and were suitable for subsequent experiments.

### Characterization of MMC and cellular activity

**Fig. 2:**
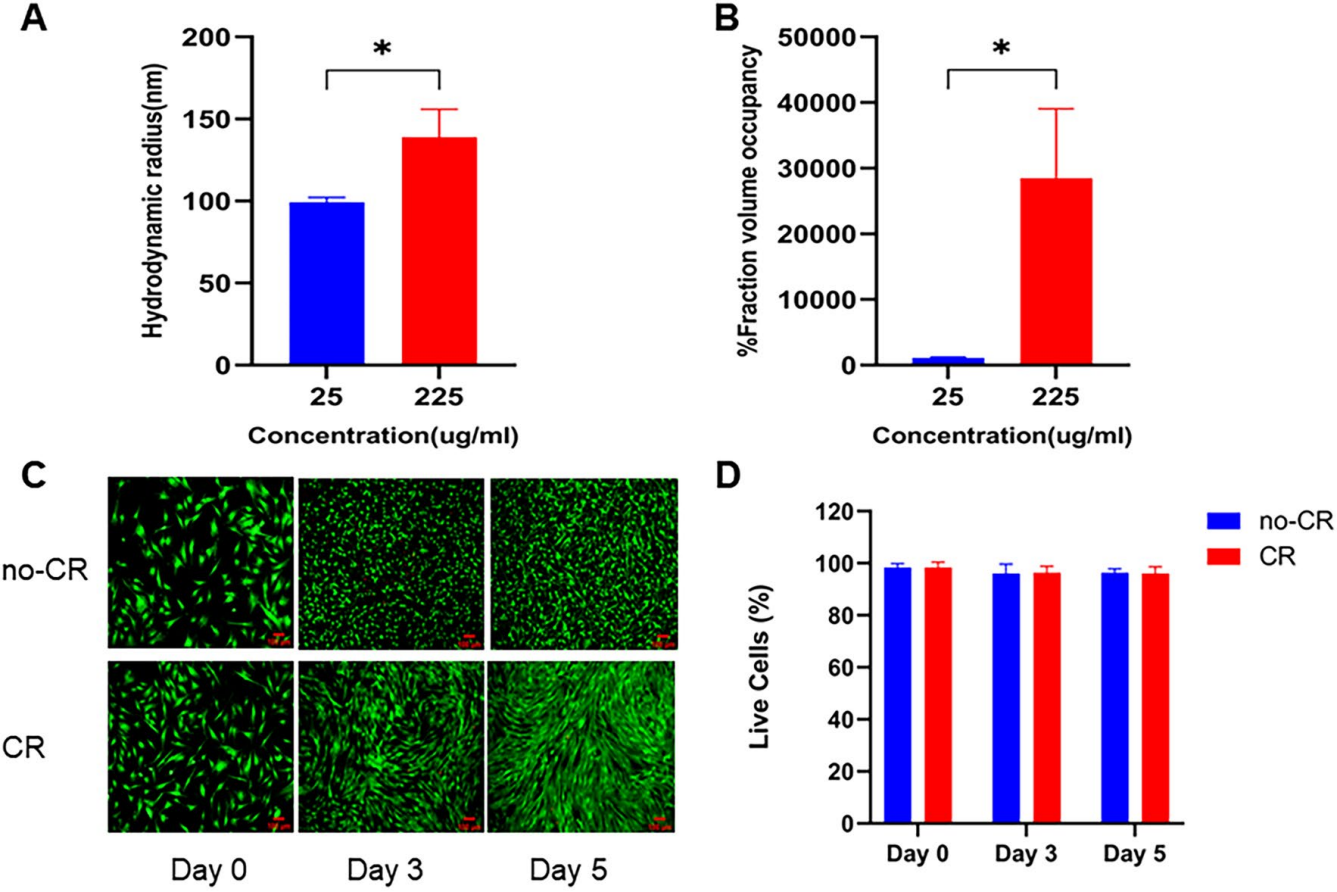
Characterization of culture environment crowding degree (**A**, **B**) and CR on cell activity (**C**, **D**). A CR significantly increased the R_H_ in the culture environment; **B** CR significantly increased FVO in the culture environment; **C** CR did not affect VSMCs survival; **D** live cell rates are all above 95%. Scale bar: 100 μm. (* indicates a significant difference from the control group, *P < 0.05)

We examined the effect of CR on cell survival and characterized the crowding in the medium after the addition of CR. The R_H_ and FVO of CR in the solution were measured and calculated using DLS. The results showed a significant increase in R_H_ and FVO in the 225 μg/mL CR group (Fig. 2A, B). Calcein/AM demonstrated that CR did not affect VSMCs survival (Fig. 2C, D).

### MMC enhances VSMCs contraction markers expression and cell contraction

**Fig. 3:**
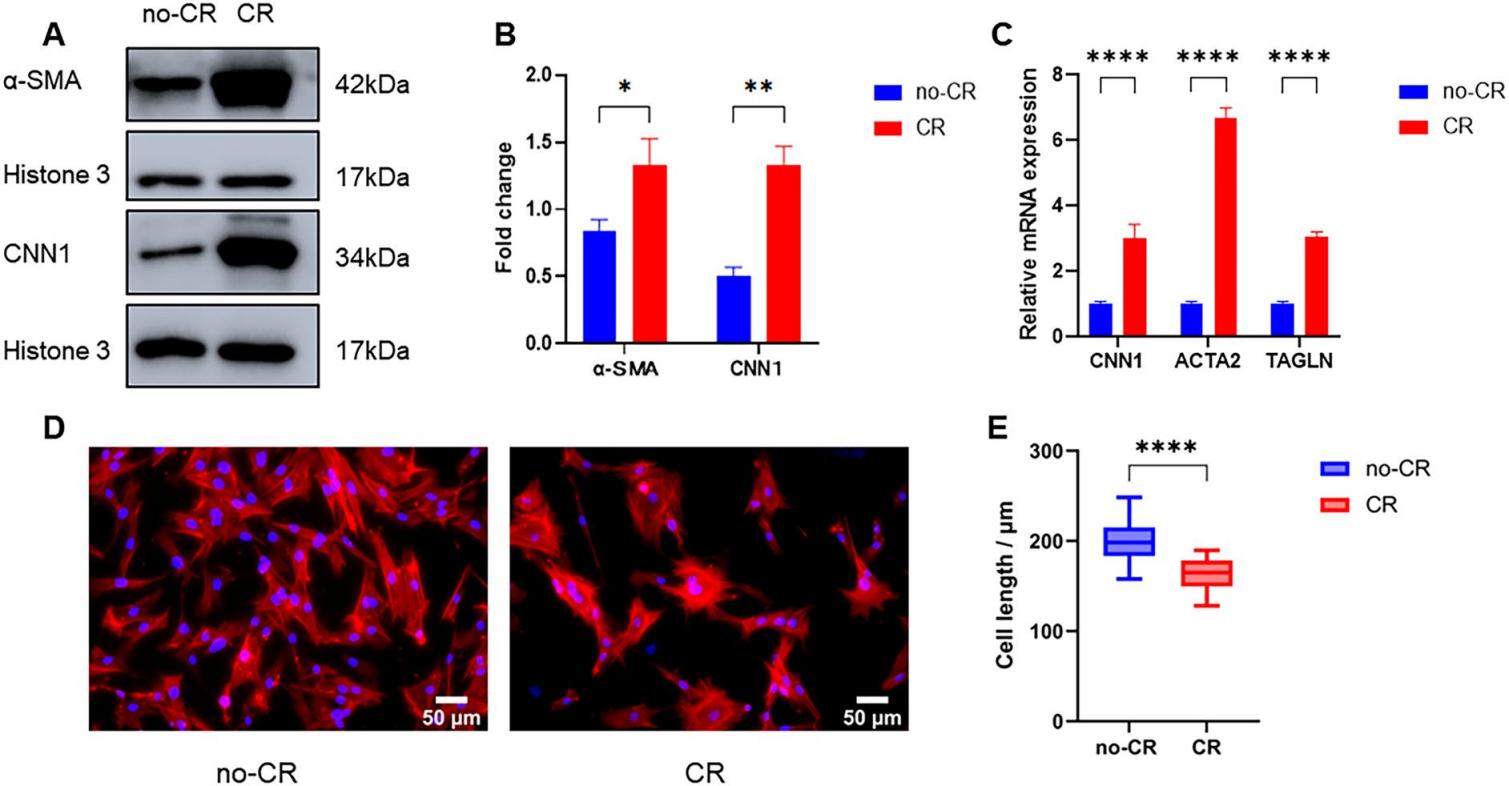
Effect of CR on VSMCs contraction markers and function (**A**–**D**). **A**, **B** VSMCs contractile phenotype markers; α-SMA and CNN1 proteins expression significantly increased in CR group; **C** VSMCs contraction markers; CNN1, ACTA2, and TAGLN genes expression significantly increased in CR group; **D** immunofluorescence staining of the VSMCs cytoskeleton in no-CR and CR group; **E** VSMCs lengths shortened in CR group. (* indicates a significant difference from the control group, *P < 0.05, **P < 0.01, ****P < 0.0001)

The effect of CR on VSMCs phenotypes was further explored by WB and RT-qPCR. After culturing for 5 d, the contractile phenotype markers α-SMA and CNN1 protein expression were significantly increased in the CR group (Fig. 3A, B). Additionally, ACTA2, CNN1, and TAGLN gene expression levels were significantly elevated (*P* < 0.05) (Fig. 3C). Meanwhile, the cell lengths were shortened in the CR group, suggesting an enhancement in cell contraction (Fig. 3D, E).

### MMC inhibits VSMCs proliferation and migration

**Fig. 4:**
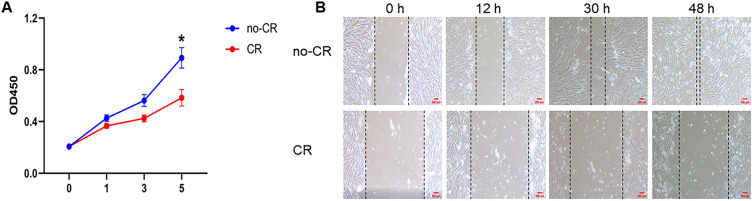
CR’s effect on VSMCs proliferation and migration (**A**, **B**). A CR significantly decreased VSMCs proliferation; B CR significantly inhibited VSMCs migration after 12, 30, and 48 h. Scale bar: 100 μm. (* indicates a significant difference from the control group, *P < 0.05)

The effect of MMC on VSMCs behavior was examined using CCK8 and cell scratch assays (Fig. 4). The results showed that MMC decreased cell proliferation, and a significant decrease in the number of VSMCs was observed on the fifth day (Fig. 4A) (*P < 0.05). VSMCs migration was significantly lower in the CR group than in the non-CR group at all time points. When the VSMCs in the no-CR group migrated to the basic fusion site, the VSMCs in the CR group remained further away (Fig. 4B).

### MMC regulates VSMCs gene expression

**Fig. 5:**
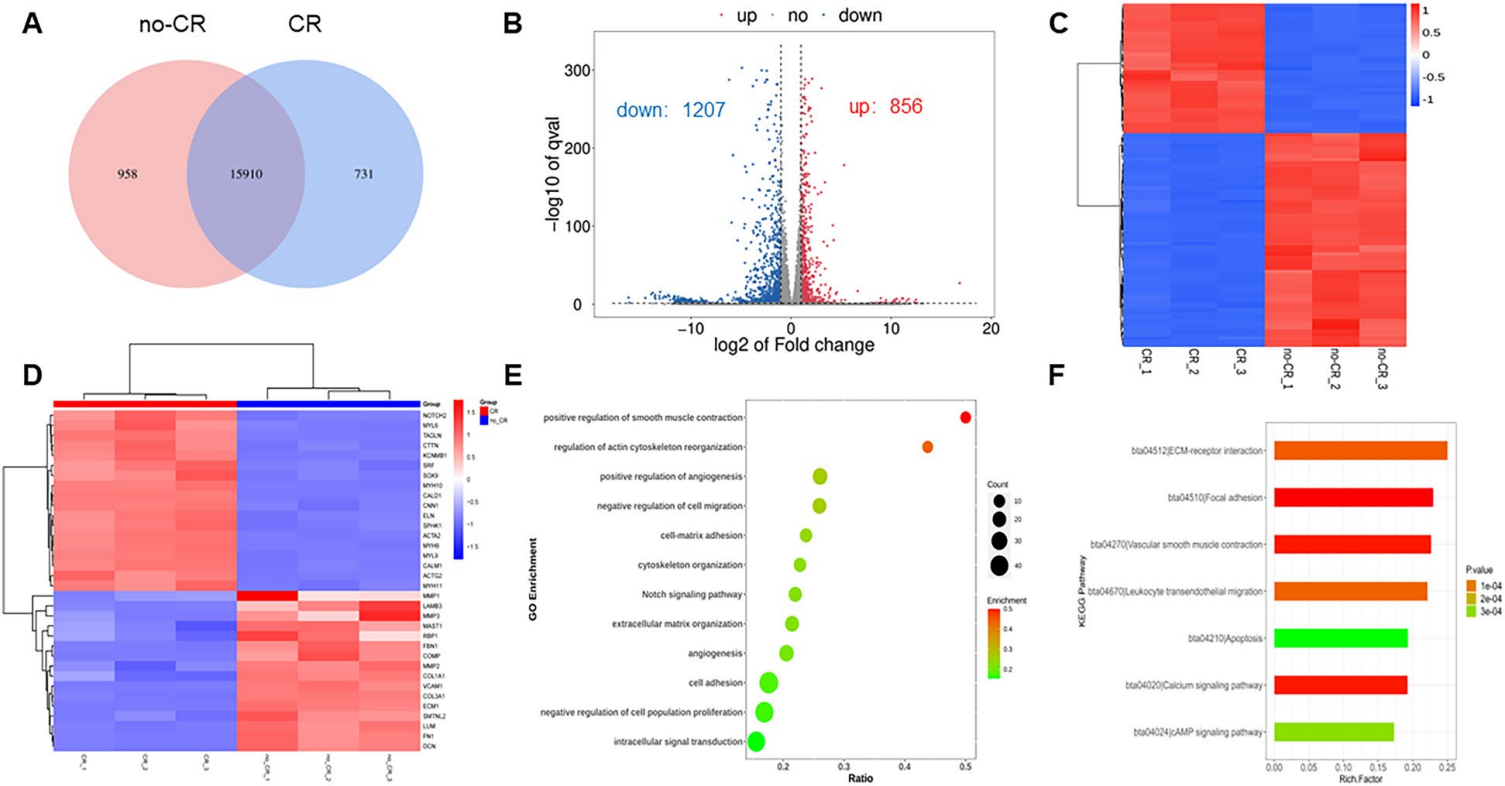
Transcriptome data analysis (**A**–**F**). **A** Genes identified in CR and no-CR group are shown in a Venn diagram; **B** volcano plot of differential genes in CR and no-CR group, red depicts upregulated genes, blue depicts downregulated genes; **C** heatmap of differential genes in CR and no-CR group; **D** heatmap of differential genes associated with contractile phenotypic behavior; **E** GO terms of differential genes associated with contractile phenotypic behavior; **F** KEGG terms of differential genes associated with contractile phenotypic behavior

**Fig. 6:**
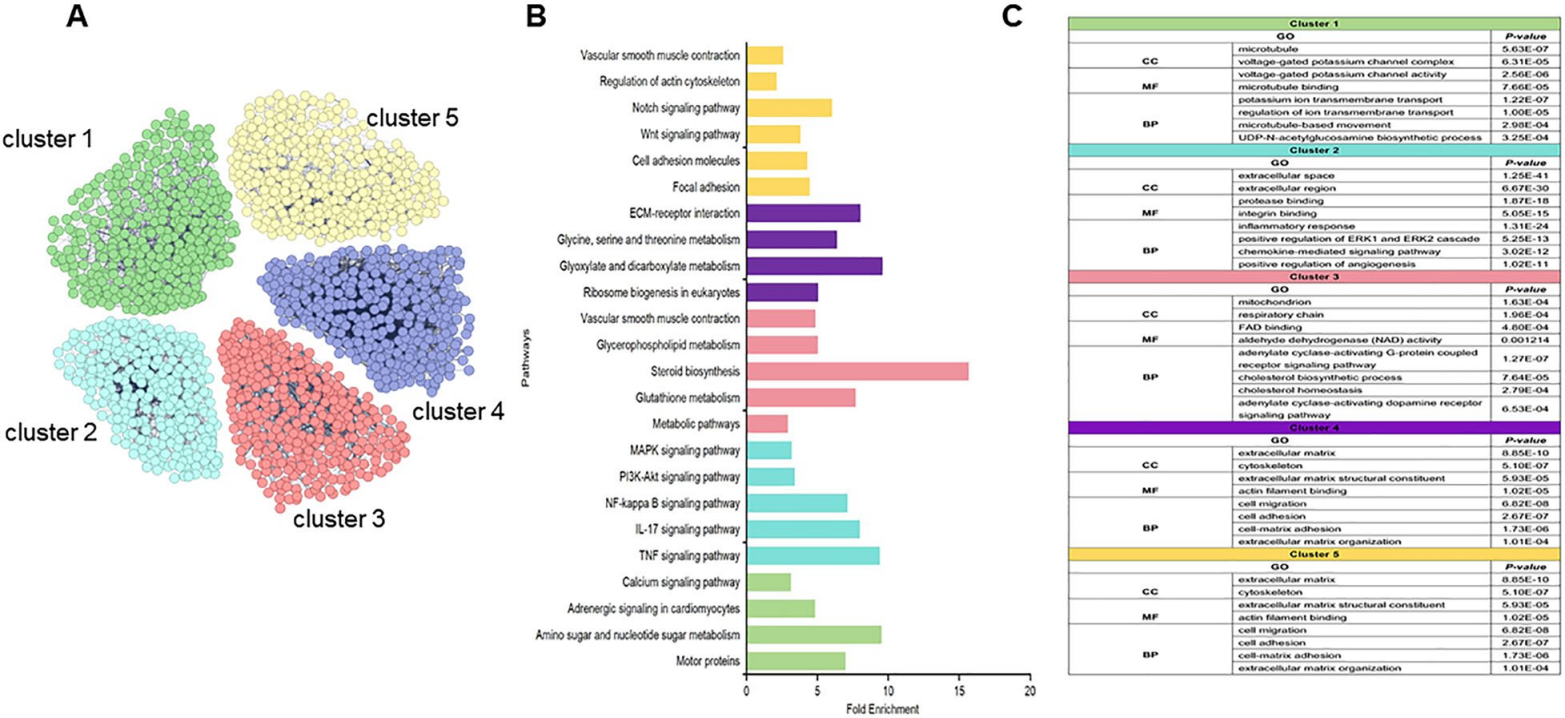
Network and cluster analysis of differential genes (**A**–**C**). A Network and cluster analysis of differential genes; B KEGG pathway terms for 5 clusters. **C** GO terms analysis for 5 clusters; CC: cellular component, MF: molecular function, BP: biological process

The effect of MMC on gene expression in the VSMCs was investigated. Gene expression in cells cultured for 5 days was detected and validated using transcriptome sequencing and RT-qPCR. In total, 958 and 731 unique genes were identified in the non-CR and CR groups, respectively. Additionally, 15,910 genes were co-expressed in both groups (Fig. 5A). Moreover, 2063 genes were screened using the threshold criteria of |log2FC|> = 1 and q < 0.05. Of these, 856 genes were upregulated and 1207 genes were downregulated (Fig. 5B). The upregulated genes were mainly concentrated in the cytoskeleton and vascular smooth muscle contraction processes, while the downregulated genes were mainly concentrated in the extracellular matrix organization and protein hydrolysis processes (Fig. 5C–F). To explore the specific functions of the differential genes, we performed protein network interaction and cluster analyses using the STRING database. Differentially expressed genes were grouped into five clusters (clusters 1–5) using the K-means method (Fig. 6A). To investigate the differential gene enrichment function and pathways in each cluster, we performed Kyoto Encyclopedia of Genes and Genomes (KEGG) (Fig. 6B) and Gene Ontology (GO) (Fig. 6C) analysis using the DAVID database. In cluster 1, GO analysis revealed that the differentially expressed genes were primarily related to ion channel complexes and microtubule movement proteins. These proteins are primarily involved in the regulation of potassium ion channels and microtubule motility. They can affect the transport of ions inside and outside the cell as well as motor proteins and calcium signaling pathways. Cluster 2 showed that genes primarily affected the binding of proteases and integrins in the extracellular space. These genes affect biological processes, such as ERK and angiogenesis, and regulation of the MAPK pathway can affect the differentiation, growth, and proliferation of VSMCs. Cluster 3 showed that the differentially expressed genes were mainly enriched in the mitochondria and affected cholesterol biosynthesis and cellular respiratory metabolism. This affects RNA and amino acid biosynthesis, as well as metabolic pathways, by regulating enzymes and proteins involved in cellular energy metabolism-related processes. Cluster 4 showed that the differential genes were mainly enriched in the nucleus and primarily involved in amino acid transport. Cluster 5 showed that the differentially expressed genes were primarily associated with the extracellular matrix (ECM) and cytoskeleton. They transport metal ions, skeleton proteins, and structural components of the ECM, and regulate cellular behaviors such as adhesion, migration, and cell–matrix adhesion. This group was enriched in several pathways, including ECM–receptor interactions, the Wnt signaling pathway, the Notch signaling pathway, VSMC contraction, and regulation of the actin cytoskeleton, which affected cell growth, development, and contraction phenotypes and functions.

**Fig. 7:**
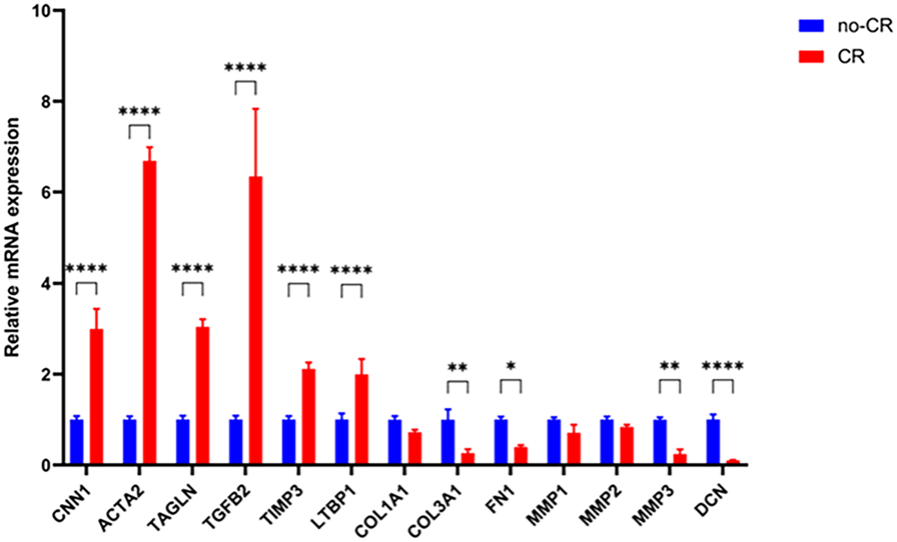
RT-qPCR of CR on gene expression in VSMCs (* indicates a significant difference from the control group, *P < 0.05, **P < 0.01, ****P < 0.0001)

Changes in gene expression confirmed that MMC affect VSMCs biological processes and signaling pathways. These changes occur mainly during cellular ion channel transport, microtubule motility, cellular respiratory metabolism, amino acid transport, cell differentiation, and ECM synthesis. Among the differentially expressed genes, we selected those related to cell growth, differentiation, and extracellular matrix for mRNA validation. RT-qPCR was used to confirm the validity of the sequencing data, which showed that the expression of these genes was consistent with the sequencing results at the mRNA level. The cell contraction phenotype (ACTA2, CNN1, and TAGLN), cell growth (TGF-B2), and TIMP3 were upregulated, whereas extracellular matrix structure and remodeling (COL1A1, COL3A1, FN1, MMP1, MMP2, MMP3, and DCN) were downregulated (Fig. 7). This suggests that MMC can regulate important genes and biological processes involved in VSMCs contractile behavior.

## Discussion

Previous studies have shown that the biological behavior of VSMCs is variable and susceptible to the culture environment [27]. Although common media have been successful for cell culture, they do not fully mimic the in vivo microenvironment required for cell growth. This can result in cell fates and behaviors that differ from those under physiological conditions [28]. We used MMC reagents to increase the macromolecular content of the medium, creating a crowded microenvironment for cell growth. This regulation of cellular biological behavior makes the in vitro environment more similar to the in vivo environment. This study is the first to regulate the VSMCs contractile phenotype and behavior using MMC. The effect of MMC on the VSMCs gene expression was comprehensively characterized using transcriptome sequencing. The results indicated that 225 μg/mL CR significantly increased the crowding degree of the medium and did not affect cell survival. Simultaneously, CR significantly promoted contraction phenotypic marker expression in VSMCs, shortened cell length, decreased cell proliferation, and inhibited cell migration. CR significantly altered gene expression in VSMCs. Specifically, 856 genes were upregulated and 1207 genes were downregulated. These alterations primarily affect the cellular ion channel transport, microtubule movement, respiratory metabolism, amino acid transport, and extracellular matrix synthesis. The upregulated genes were primarily involved in the cytoskeleton and the contraction processes of VSMCs, whereas the downregulated genes were mainly involved in extracellular matrix synthesis. Previous studies have explored the effect of MMC on fibroblast and stem cell behaviors [16–18]. Our results elucidate the effect of MMC on VSMCs behaviors and reiterate that MMC does not affect cell survival, but affects cell proliferation and migration capacity and regulates cell phenotypes.

DLS measurements of 10 μg/mL CR were below the instrument's lower detection limit, resulting in failure to measure [23]. Therefore, in this study, 25 μg/mL CR was used as the reference concentration to compare the crowded environments produced by different concentrations of CR (Fig. 2A, B). The R_H_ is a crucial metric for characterizing and predicting molecular crowding in aqueous physiological environments. This represents the effective size of crowded molecules. FVO can approximate the degree of crowding produced by crowded molecules in a culture environment and can be calculated from the R_H_ [12]. Although electrostatic repulsion and hydration may enhance the excluded volume effect (EVE), the resulting FVO may be lower than the actual crowding capacity; therefore, we chose to neglect this effect of the resulting crowding. This is because it is difficult to estimate the unavailable space accurately, making it challenging to compute the total EVE. Therefore, we used R_H_ and FVO as the primary metrics to assess the degree of crowding caused by CR [25]. It had been demonstrated that FVO increased exponentially with concentration of 10–500 μg/mL CR, resulting in significant crowding. Concentrations exceeding 500 μg/mL altered the viscosity of the medium and were not utilized. We selected an intermediate concentration of 250 μg/mL CR for our study. To ensure sterility, the medium containing CR was filtered using a 0.45-μm sterile filter membrane and prepared with a sterile medium containing 10% FBS. The final concentration of the medium was 225 μg/mL CR. Consistent with previous studies [23], our results showed that 225 μg/mL CR significantly increased crowding degree in solution, providing a crowded culture environment for cell growth in vitro. Importantly, this concentration did not affect cell survival, suggesting its potential for use in cell culture (Fig. 2C, D).

The VSMCs culture in MMC medium showed noticeable phenotypic and behavioral changes. The VSMCs contractile phenotype markers were upregulated and cell lengths were shortened (Fig. 3), suggesting that MMC reverted VSMCs to a contractile phenotype in vitro in a physiological state. This contributed to VSMCs performing physiological functions close to their in vivo counterparts. The expression of contractile markers is regulated by various factors, including the growth environment, cell density, and gene regulation [29]. However, the mechanism underlying MMC-mediated VSMCs contractile phenotype remains unclear. Transcriptome sequencing suggested that MMC regulates the expression of contractile phenotype marker proteins by upregulating gene expression and downregulating extracellular matrix gene expression. This was consistent with the contractile cellular phenotype (Fig. 5D). Secondly, MMC could affect the integrative mechanisms of cellular adaptation by altering the arrangement of the cytoskeleton or the expression of microtubule proteins. This could lead to remodeling of the microtubule cytoskeleton, changes in intracellular trafficking, and alterations in cellular gene expression that regulate cellular function, tissue development, and regeneration (Fig. 5E) [16, 30]. In this case, alterations in the VSMCs contractile pathway were accompanied by changes in the calcium ion signaling pathway. It was speculated that MMC may regulate VSMCs contractile function by affecting the calcium ion signaling pathway, and that the inward flow of calcium ions enhances cell contractile function (Fig. 5F) [31]. MMC significantly inhibited VSMCs proliferation and migration (Fig. 4). This inhibitory phenomenon is the main behavioral manifestation of the VSMCs contractile phenotype. Previous studies had reported a decrease in cell proliferation, which may be due to MMC inducing cellular gene transcription that alters the number of cells [18, 32]; VSMCs migration is crucial for various physiological processes, including development, immune defense, and wound healing. Cells use different migration modes in complex natural environments, which are regulated by the extracellular microenvironment, enzymatic signaling, and myosin contractility [33]. For most cell types, integrin receptors establish direct contact with extracellular matrix proteins such as collagen or fibronectin [34, 35]. The decrease in VSMCs migration may be attributed to MMC, which affects the kinetics and stability of actin polymerization, leading to cytoskeleton impairment and reduced cellular drive [36]. Transcriptomics suggested that MMC significantly affects microtubule movement, integrin-based extracellular matrix, and cytoskeleton-related biological processes and pathways. Additionally, MMC may decrease cell migration ability by affecting gene expression (Fig. 6). Furthermore, ECM deposition increases cell–substrate adsorption, which in turn decreases cell migration (Fig. 4) [16, 37]. These alterations in VSMC phenotypes and behaviors will have wide-ranging implications for tissue engineering and regenerative medicine. Ideal tissue-engineered vascular grafts require the synergistic action of ECM and VSMCs, and the use of CR or similar preparations to create scaffolding materials that support the contractile phenotype can enhance the mechanical properties of the grafts and facilitate the development of vascular grafts and tissue repair strategies [38]. This could also be achieved by comparison with in vivo VSMC behaviors, especially in vascular disease states such as hypertension or atherosclerosis. This will help validate the physiological relevance of the findings, as well as identify any differences or limitations of the in vitro model, facilitating the study of the mechanisms of transformation of VSMC performance and the pathogenesis of vascular disease.

To comprehensively analyze the behavior and phenotype of VSMCs, we used transcriptomic sequencing to detect and analyze gene expression. A distinguishing feature of cells cultured in vivo and in vitro is the density of the molecules surrounding the cell, which plays a crucial role in regulating cellular dynamics. Molecular crowding is a natural state in which the microenvironment enabling cell growth is crowded with macromolecules. Molecular crowding induces EVE, which reduces the diffusion efficiency and enhances the binding rate of macromolecules. This phenomenon can significantly affect biochemical dynamics and has a fundamental impact on cellular functions such as transcriptional processes and nuclear structure [39]. However, few studies have comprehensively characterized the effects of MMCs on the gene expression in VSMCs. Therefore, this study explored the differences in gene expression between VSMCs cultured in MMC and those cultured in non-MMCs using transcriptome sequencing and bioinformatic analysis. This study revealed that MMC significantly altered the transcriptional processes and gene expression. It has been hypothesized that alterations in gene expression directly affect the fate and function of VSMCs. Cluster analysis indicated that VSMCs in the two culture environments differed in several cellular processes including ion channel transport, microtubule movement, cellular respiration and metabolism, amino acid transport, extracellular matrix protein synthesis, and growth and development (Fig. 6). Cluster 1 demonstrated that MMC affect ion channel complexes and microtubule movement. Calcium ions play a crucial role as secondary messengers in VSMCs. VSMCs regulate intracellular calcium ion concentrations by expressing calcium channels and transporter proteins, which control VSMCs contraction, proliferation, and migration. Therefore, we hypothesized that this may affect cell contractility. MMC might increase the intracellular calcium ion concentration, promoting VSMCs’ contractile phenotype and contractile ability [40]; Microtubules are highly dynamic structures that undergo repetitive cycles of polymerization and depolymerization. They were fine-tuned to meet the needs of cells and tissues. The ability of microtubules to regulate cell migration through coordinated interactions between polymerization/depolymerization and adhesion patch assembly/disassembly, as well as their ability to regulate Rho-GTPase signaling for actin contractility, suggests that the changes in microtubule motility could account for alterations in the migratory and contractile capacities of cells [41, 42]. Clusters 2 and 5 were both biological processes that affected the extracellular space but the focus was different; cluster 2 primarily affected integrin signaling and the differentiation and growth of VSMCs, and cluster 5 focused on extracellular matrix proteins and cytoskeletal processes that regulated cellular behaviors. The results showed that the MMC could significantly affect cell migration, adhesion and cell–matrix interactions, which was consistent with our observed cellular behaviors, suggesting that MMC might alter the VSMCs gene expression to affect cellular behaviors; secondly, the transcriptomics sequencing demonstrated that the expression of most of the ECM genes was downregulated (Fig. 5D), which was also consistent with the VSMCs contractile phenotype. ECM deposition increased in previous studies, which further validated that ECM increase was mainly a physical process rather than a gene-regulated biological process. The gene expression was downregulated in MMC, but ECM deposition was increased, which also suggested that the ECM synthesized by the cells was lost in the medium in large quantities. Thus, reducing the loss of ECM in the medium also needs further exploration [18, 37, 43]. Clusters 3 and 4 are intracellular organelles that regulate mitochondrial and cellular metabolism, RNA and amino acid biosynthesis, and metabolic pathways, consistent with the protein levels that had been reported in the literature [44]. This affects the overall rate of protein synthesis, which is a hallmark of cell and tissue growth. These mechanisms were not explored in depth in our findings. We speculate that the following molecular mechanisms may contribute to the maintenance of the contractile phenotype in VSMCs. MMC may regulate intracellular calcium ion concentration by affecting calcium ion channels or calcium ion pumps in VSMCs, affecting the contractility of VSMC. It may affect the role of transcription factors associated with VSMC contraction and cytoskeletal remodeling. MMC also may affect the activation state of RhoA/ROCK, MAPK and TGF-β signaling pathways activation status, regulating or maintaining VSMCs contractility and phenotype. VSMCs phenotypic shifts play a key role in cardiovascular diseases such as hypertension and atherosclerosis [2, 27]. Thus, MMC could alter the VSMCs contractile phenotype and behavior by affecting cellular gene expression, providing fundamental data for subsequent in-depth regulation of the molecular mechanisms that alter the contractile phenotype and behavior, as well as providing a cellular model for the study of the pathogenesis of vascular diseases, such as hypertension.

This study comprehensively characterized the contractile phenotypic behaviors and gene expression of VSMCs cultured in MMC using protein analysis and transcriptome sequencing, which provided a reference method to regulate the VSMCs contractile phenotype and behaviors in vitro, as well as basic data for subsequent in-depth investigation of the molecular mechanisms. This study also has some limitations. First, the in vitro model cannot fully simulate the in vivo environment. Furthermore the potential effects of different concentrations of CR, as well as the long-term stability of CR’s effects of CR on VSMC are still unknown. Moreover, whether CR produces effects other than increasing medium crowding, in vivo validation, still needs to be further explored. In the future, CR concentrations and VSMC phenotypic shifts at different time points for assessing the persistent role of MMC, such as CR, in maintaining the contractile phenotype, as well as the application of CR in a 3D culture system or its interactions with other microenvironmental factors to better mimic the in vivo environment should be studied.

This study focuses on the specific molecular mechanism by which MMC affects the VSMCs contractile phenotype, behaviors, and functions. It also comprehensively characterizes the differences in protein expression by combining proteomics with transcriptomics technology, to discover the relationship between gene and protein levels, and to accelerate the construction of the contractile phenotype and maintain the stability of the VSMCs contractile phenotype. This not only provides an excellent cellular model for the study of vascular diseases such as hypertension, but also contributes to the development of new therapies for vascular diseases that target the maintenance of the vascular smooth muscle cell phenotype by modulating the microenvironment in order to prevent or reverse pathological changes in VSMC.

## Conclusion

In this study, we constructed the MMC environment by adding CR to increase the medium crowding degree without affecting cell survival, and found that MMC could promote contractile phenotypes and behaviors in VSMCs, such as increase in contractile phenotypic markers, shortening of cell lengths, and inhibition of cell proliferation and migration, and then comprehensively characterized genomic changes in VSMCs under MMC by using transcriptome sequencing technology. The changes mainly occurred in cellular ion channel transport, microtubule movement, cellular respiration and metabolism, amino acid transport, and extracellular matrix synthesis. The results showed upregulated genes mainly in the cytoskeleton and the process of vascular smooth muscle contraction, and the downregulated genes mainly in the process of extracellular matrix synthesis. These phenomena are consistent with the contractile phenotype of VSMCs. This study provides a reference for constructing the VSMCs contractile phenotype in vitro, which can provide an excellent cell model for the study of vascular diseases such as hypertension.

## Data Availability

Not applicable.

## Abbreviations

VSMCs: Vascular smooth muscle cells
MMC: Macromolecular crowding
CR: Carrageenan
MYH11: Smooth muscle myosin heavy chain
ACTA2: Smooth muscle alpha-actin
TAGLN: Sm22 alpha
MYOCD: Myocardin
H3: Histone 3
2D: Two-dimensional
FBS: Fetal bovine serum
D × S: Dextran sulfate
CNN1: Calponin
GAGs: Glycosaminoglycans
R_H_: Hydrodynamic radius
FVO: Volume occupancy fraction
DLS: Dynamic light scattering
EVE: Excluded volume effect
ECM: Extracellular matrix
